# Supplementary material for: The inflammatory response of human pancreatic cancer samples compared to normal controls
Source: PLoS One. 2023 Nov 1;18(11):e0284232. doi: 10.1371/journal.pone.0284232 (PMC10619777; doi:10.1371/journal.pone.0284232)
Supplement: S1 Appendix — S1 Fig. The heatmap summarizes the supervised clustering and differential gene expression analysis comparing the normal samples to the tumor samples. Dendrograms show the unsupervised hierarchical clustering of samples(top) and genes (left side). S2 Fig. CNET plot showing gene overlaps in enriched genesets. Geneset enrichment analysis of genes differentially expressed between tumor andnormal samples indicated enrichment for sets of genes involved in KRAS signaling (purple), inflammatory response (blue), complement cascade (green),and epithelial mesenchymal transition (turquoise). Figure also includes genes known to up (orange) or down (olive) regulated in PDAC. Black dashed circle indicates PLAUR, which connects to all 5 of the 6 genesets (not connected to down in PDAC). Genes at the end spokes indicate differential expressed inour dataset that were enriched in each geneset, color of dots indicated whether the gene was up (red tones) or down (blue tones) regulated in tumors relative to normal samples [17, 21, 36]. (PDF) [file pone.0284232.s001.pdf]

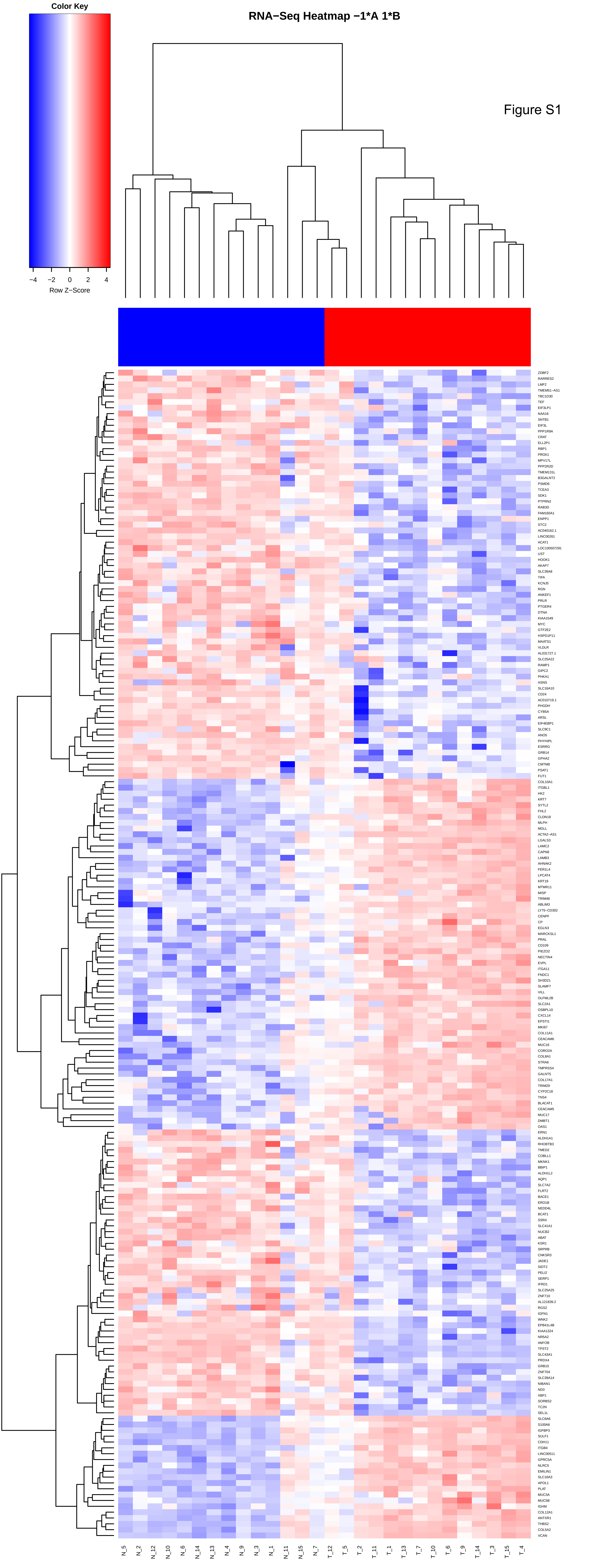


Figure S1

Figure S2:

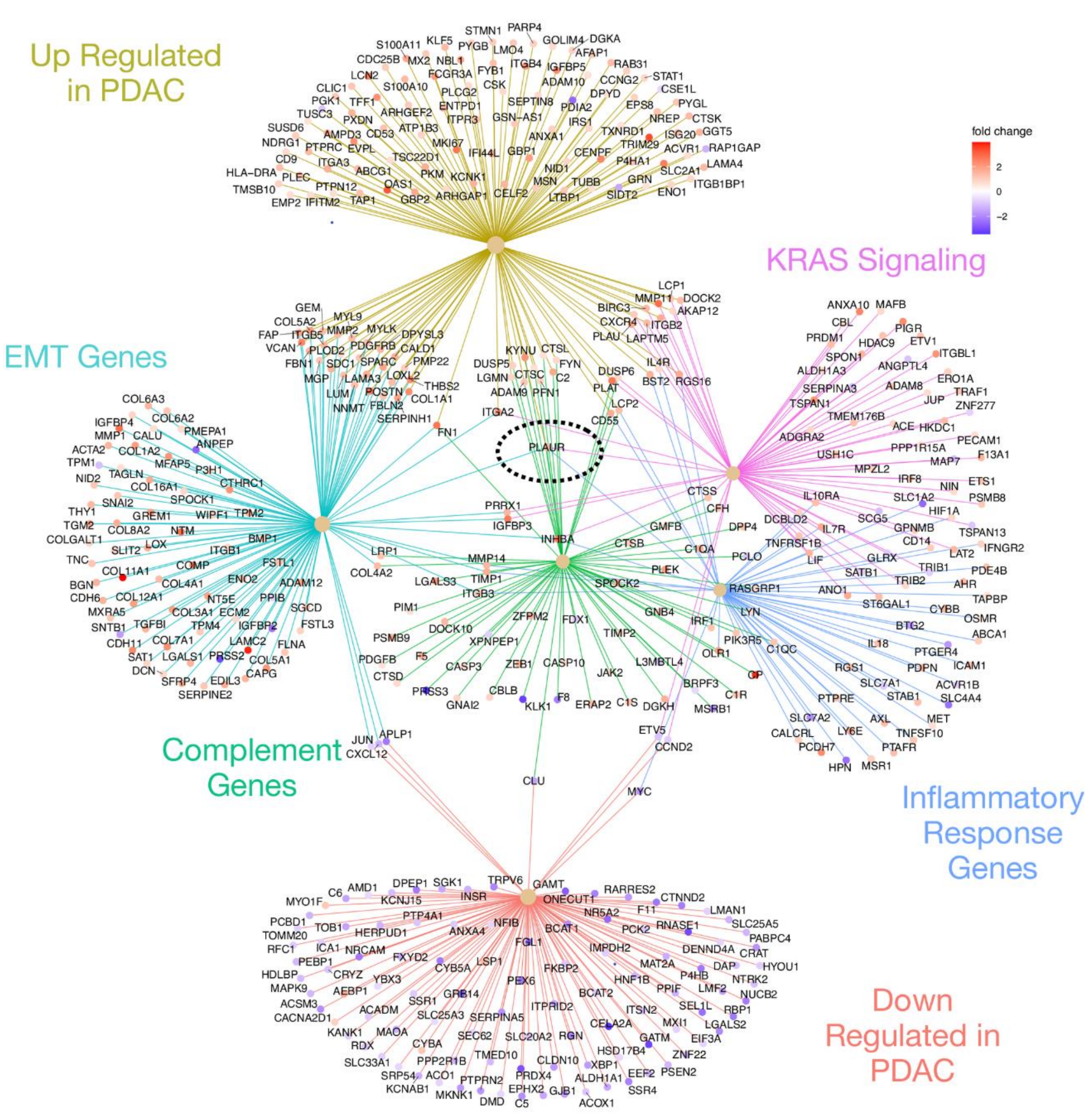


Fig S1: The heatmap summarizes the supervised clustering and differential gene expression analysis comparing the normal samples to the tumor samples. Dendrograms show the unsupervised hierarchical clustering of samples(top) and genes (left side).

Fig S2: CNET plot showing gene overlaps in enriched genesets. Geneset enrichment analysis of genes differentially expressed between tumor andnormal samples indicated enrichment for sets of genes involved in KRAS signaling (purple), inflammatory response (blue), complement cascade (green),and epithelial mesenchymal transition (turquoise). Figure also includes genes known to up (orange) or down (olive) regulated in PDAC. Black dashed circle indicates PLAUR, which connects to all 5 of the 6 genesets (not connected to down in PDAC). Genes at the end spokes indicate differential expressed inour dataset that were enriched in each geneset, color of dots indicated whether the gene was up (red tones) or down (blue tones) regulated in tumors relative to normal samples. (17, 21, 36).
