# Supplementary material for: The inflammatory response of human pancreatic cancer samples compared to normal controls
Source: PLoS One. 2023 Nov 1;18(11):e0284232. doi: 10.1371/journal.pone.0284232 (PMC10619777; doi:10.1371/journal.pone.0284232)
Supplement: Attachment — Submitted filename: Response to Reviewers-fungous-PDAC.docx [file pone.0284232.s002.docx]

**Response to Reviewers**

1. 14 samples, even though they are paired might be too low of a number, are you sure you have enough power to do the study? You could also study genes from the TCGA database besides your own data set and the ones from Gruetzmann dataset.

**Answer**:

1. A UNM statistician felt that tools like DeSeq2 and EdgeR are specifically designed to analyze RNA-seq counts, even with low sample sizes. They have consistently identified differential expression and pathways in previous cases with smaller sizes with unpaired samples. Our application of these tools successfully detected differentially expressed genes even after multiple hypothesis correction, which is statistically sound.

a. We used the Grützmann data set as referenced in ref (20)

Manuscript' line 140

'Revised Manuscript with Track Changes Line 150

b	We used EdgR ref (34, 35)

Manuscript line 268

‘Revised Manuscript with Track Changes line 280

c	We used DeSeq2 ref (34)

Manuscript line 265

‘Revised Manuscript with Track Changes line 282

2. Line 53: how much longer specifically did those patients live who had a richer gut microbial community.

**Answer**: “. Patients with high diversity and low diversity had overall survival of 9.66 vs. 1.66 years respectively using univariate Cox proportional hazard models”. This has been added to the manuscript.

Manuscript' Line 56

'Revised Manuscript with Track Changes Line 56

3. Lines 103-106: 3,177 genes is quite a large number to find. Did you use Benjamini-Hochberg correction? The number of false positive genes might be too high.

**Answer**: Yes. We did use the Benjamini-Hochberg (BH) correction.

“As shown in Figure 1B, we found 3,177 genes that were at least 1.5-fold up or down regulated (up = 1593, down = 1584) and had a p-value (Benjamini-Hochberg adjusted) less than or equal to 0.05.”

Manuscript'	Line 111:

'Revised Manuscript with Track Changes: Line 112:

4. Line 250: Why did you not use DESeq2?

**Answer**: We used EdgeR which in our opinion is a perfectly acceptable alternative tool. However we also used DESeq2. We realized that the methods section says DESeq, and not DESeq2. We corrected the typo to accurately reflect our use of DESeq2 and not DESeq.

Manuscript: Line 265

'Revised Manuscript with Track Changes Line 281

5. Line 254; in what way did you use clusterProfiler? Since you used these tools to generate results, it is essential to document their use.

**Answer**: ClusterProfiler, an R package, was used to analyze the differentially expressed genes for gene set enrichment (GSEA) against the Molecular Signatures Database and visualize differentially expressed genes in gene sets of interest (16, 17, 36). Default values were used at all points.

Manuscript Line 270:

'Revised Manuscript with Track Changes: Line 290

6. Line 176-177: the term “evolutionarily conserved” is a very poor choice of terms. I suggest not using it, since it is an oxymoron. In a broad sense, evolution means change, but conservation denotes stasis. The term evolutionary conservation implies something changing while standing still. 'Revised Manuscript with Track Changes

**Answer:** We removed the word “evolutionary” from the manuscript

Manuscript Line 187

'Revised Manuscript with Track Changes Line 198

7. Line 195: “β-glucans is a major cell wall component of fungi”, this is an orphan sentence, why is this statement relevant to your findings?

**Answer**: We removed the sentence from the manuscript.

Manuscript Line 203

'Revised Manuscript with Track Changes Line 217

8. Lines 205-206: please add a reference supporting the claim that C2 has a role in BCR signal transduction.

**Answer**: We placed a reference (22) with some discussion supporting the claim that C2 has a role in BCR signal transduction.

Manuscript Line 177 ref (22), line 170 (discussion)

'Revised Manuscript with Track Changes Line 147 reference (22), 140 (discussion)

9. Line 219: do you have clinical results from similar studies?

**Answer**: We added data of 2 clinic papers supporting the effect of galectin-3 and PLAU on PDCA prognosis.

Examples of clinical correlation between PDCA prognosis and these genes include: 1) A correlation was noted between the overall survival of the pancreatic cancer patients with plasma Galectin-3 levels (n=21). The high-galectin-3 group (n=10) showed a poorer prognosis than the low-galectin-3 group (n=11) in pancreatic cancer patients (p=0.006). (ref 29), and 2), PLAU upregulation was associated with the basal type of PDAC, and an increased PLAU protein expression is associated with poor prognosis (P=0044), and within the basal subtype, the clinical outcome in the high PLAU expression group is significantly worse than the low PLAU expression group (P=0.018). (ref 30).

Manuscript Line 229

'Revised Manuscript with Track Changes Line 240

10. Lines 222-227: you state that there was no difference in the Malassezia between tumor and non-tumor samples. Why then do you think that altering the microbial flora around and in the tumor will have a therapeutic effect?

**Answer**: We removed the sentence “The role of the microbiome, including fungi is being recognized as having a significant role in the pathophysiology and progression of PDAC, and we believe solving how to alter the flora around and in the tumor can add another dimension to treating this disease” We are not going to comment about the microbiome with respect to outcome of PDCA, as our data does not address this issue.

Manuscript Line 240

'Revised Manuscript with Track Changes Line 254

**Journal Requirements**

1. We have adapted the PLOS ONE’s style requirements
2. We have removed funding acknowledgments from the manuscript, and have added: “The funders had no role in study design, data collection and analysis, decision to publish, or preparation of the manuscript” to the cover letter

3. We have added: “I have read the journal's policy and the authors of this manuscript have the following competing interests: NONE” in the cover letter.

4. In your Data Availability statement, you have not specified where the minimal data set underlying the results described in your manuscript can be found. PLOS defines a study's minimal data set as the underlying data used to reach the conclusions drawn in the manuscript and any additional data required to replicate the reported study findings in their entirety. All PLOS journals require that the minimal data set be made fully available. For more information about our data policy,

Genomics Shared Resource at the UNM Comprehensive Cancer Center. RNA sequencing data is available for download from the NCBI BioProject database using study accession number PRJNA940178. However, this will be made available to review once the paper has been accepted.

5. Please include your full ethics statement in the ‘Methods’ section of your manuscript file. In your statement, please include the full name of the IRB or ethics committee who approved or waived your study, as well as whether or not you obtained informed written or verbal consent. If consent was waived for your study, please include this information in your statement as well.

We have added the full ethics statement in the Methods section

6. Please upload a new copy of Figures 1, 2, 3, 4, 5 and 6 as the detail is not clear. Please follow the link for more information

We improved the figures 1,2,3,4,5, and 6 firstly by taking them out their ”boxes”, and displayed them one by one, in the manuscript. We will also send a copy of the figures independently, which have better visualization characteristics. Figure 2 C is still not perfect but is fine in the attached copies of the figures.

7. Please include a new copy of Table 2 in your manuscript; the current table is difficult to read.

We have corrected table 2 (now table 3)

8. Please include your tables as part of your main manuscript and remove the individual files. Please note that supplementary tables should be uploaded as separate "supporting information" files.

We put the three tables in the main manuscript and the Heat map and CNET plots supplementary figures in the supporting information.

**Additional Reviewers Questions**

1- The PDAC RNA-seq summary (Table1) is based on normalized count data and after filtration? Please add more details at the caption along with total number of genes remain after filtration. This table shows raw, unfiltered and un-normalized sequencing data statistics. The caption was edited to include additional information to clarify this point as well as how the information was determined.

2- I assume authors considered the same format of data for all the analysis including filtered normalized data.
a. As an example, for Figure 3 & 6, it was mentioned log2 count expression. So, do authors keep working on log2 normalized RNA-seq (after filtration)? Please clarify this part. Updated
3- Figure 1A shows the MDS analysis. The heterogeneity across tumor samples can be related to the tumor sub-types. Is there any data information about the sub-type of PDAC data? If yes, that’s great to include sub-type info in MDS analyses. No
4- Table 2 represents the pathway enrichment analysis while the column between p value and q value is not clear. Please keep consistency about the multiple test correction approach and its notation, qvalue, adjusted p-value, or BH? Fixed. Also fixed to indicate definition of GeneRatio and BgRatio
5- Method section needs to be improved Updated
a. edgeR package includes different normalization method. Which one was applied for data? Clarified
b. MDS or PCA was applied? Clarified
c. I couldn’t follow why and where DESeq2 was applied? Clarified
d. How authors include the paired sample differentially expressed analysis using edgeR package? Corrected misleading terminology. 'Paired' was replaced with 'matched' to indicate that both the tumor and normal samples came from the same patient, however to analysis was not a paired analysis.

e. Please add other analysis were used in the manuscript including GSEA
f. I assume heatmaps show unsupervised clustering (e.g., SFig1)? No, as stated in the figure legend this heatmap is a supervised heatmap.
g. Boxplots (Fig 3 & 6) show considerable differences between tumor and normal. It is great to choose an appropriate statistical method and add the p-value on each figure. Added
h. Add the type of multiple test correction approach that was applied across manuscript and potential cut-off (for all analysis). Updated
6- Authors did findings based on only one PDAC cohort. Did authors try any other cohorts using the same analysis to see if it is possible to generalize their findings? No
a. What are the main limitations of this research study? This cohort has few samples. Is there any treatment response data for the samples or any other molecular data (DNA)? This a small sample. The samples were anonymized, and thus there was no clinical data to review. There is PDCA prognosis data in humans for gal-3 and UPAR that is noted in the manuscript.

b. Also is it possible to share how can get access to this cohort? Unfortunately no
7- Figures have low resolutions and need to be improved.
a. As an example, the heatmaps are not clear. done
b. Please add on the caption, the number of genes included in heatmaps or volcano, and MDS plot done
c. Y-axis at the volcano plot shows the -log10 p-value (where the p-value shows adjusted p-value or BH?). The figure needs to be improved. done
i. Y-axis can be -log10 p-value

- A rebuttal letter that responds to each point raised by the academic editor and reviewer(s). You should upload this letter as a separate file labeled 'Response to Reviewers'.
- A marked-up copy of your manuscript that highlights changes made to the original version. You should upload this as a separate file labeled 'Revised Manuscript with Track Changes'.
- An unmarked version of your revised paper without tracked changes. You should upload this as a separate file labeled 'Manuscript'.
