# Supplementary material for: The inflammatory response of human pancreatic cancer samples compared to normal controls
Source: PLoS One. 2023 Nov 1;18(11):e0284232. doi: 10.1371/journal.pone.0284232 (PMC10619777; doi:10.1371/journal.pone.0284232)
Supplement: Attachment — Submitted filename: Rebuttal Letter of Response.docx [file pone.0284232.s003.docx]

**Letter of Response to the academic editor and reviewers. 8/24/2023**

PONE-D-23-09005R1

Case 08128318
PL#0N3_AR
ref:_00DU0Ifis._500PMn9N8:ref

Major Concern: The changes of inflammation may not reflect the presence or absence of the fungus but could represent the tumor itself.

**Answer**

Line 1 new	line 1 tracked

We agree with this sentiment and have made changes to the manuscript.

a. We have changed the title of manuscript from:

“The immune response to a fungus in pancreatic cancer samples”

To:

“The inflammatory response of human pancreatic cancer samples compared to normal controls”

b. Line 250 new	tracked version 250

We added this sentence in the discussion.

“Based on our data we have no way of determining if this is a reaction to a fungus or the tumor itself.”

Comments to Author

**Answer**

1. Both Reviewers said “all comments have been addressed”
2. Is the manuscript technically sound and do the data support the conclusion
a. Reviewer #1 said partly, and Reviewer #2 said yes

**Answer**

It is unclear what the “partly” refers to, but we have addressed a number of issues in this response letter.

3. Has the Statistical analysis been performed appropriately and rigorously

**Answer**

Reviewer #1 said “I don’t know”, reviewer #2 said yes.

To answer Reviewer # 1: There is an overlap of question 3 and 6.

For #3: As we have mentioned before: We have added a similar passage into the manuscript (in lines 244-250 new and 244-250 tracked versions) for question #6

This was a single institution study, and we got the samples from the Hospital Tissue Bank. A UNM statistician felt that tools like DeSeq2 and EdgeR are specifically designed to analyze RNA-seq counts, even with low sample sizes. They have consistently identified differential expression and pathways in previous cases with smaller sizes with unpaired samples. Our application of these tools successfully detected differentially expressed genes even after multiple hypothesis correction, which he believed is statistically sound.

4. Data of findings of the manuscript fully available

**Answer**

Both the reviewers said yes

5. The manuscript was presented in an intelligible fashion and written in standard English

**Answer**

a. Both reviewers said yes
6. Review comments to the Author
a. Reviewer # 1. (No Response)

Reviewer # 2 said the study does not have enough power to generalize the results due to a limited number of samples

**Answer**

This issue is addressed on pages.

Line 244-50 new and tracked lines 244-250 versions

This was a single institution study, with only 14 samples from the Hospital Tissue Bank. It was felt that tools like DeSeq2 and EdgeR are specifically designed to analyze RNA-seq counts, even with low sample sizes. They have consistently identified differential expression and pathways in previous cases with smaller sizes with unpaired samples. Our application of these tools successfully detected differentially expressed genes even after multiple hypothesis correction. However, we acknowledge that this may not have enough power to generalize the results.

Sincerely

Ian Rabinowitz
